# Supplementary material for: High Throughput Screening Targeting the Dengue NS3-NS5 Interface Identifies Antivirals against Dengue, Zika and West Nile Viruses
Source: Cells. 2022 Feb 18;11(4):730. doi: 10.3390/cells11040730 (PMC8870125; doi:10.3390/cells11040730)
Supplement: Supplementary file 1 [file cells-11-00730-s001.zip › cells-1550383-supplementary.pdf]

Supplementary Materials

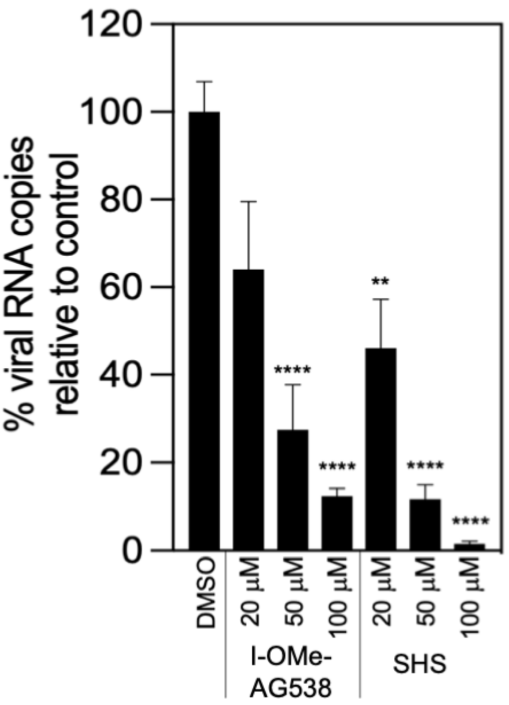

Figure S1. I-OMe-AG538 and SHS inhibit DENV-2 replication. Vero cells were pretreated for 2h with or without the indicated concentrations of compound, and then infected with DENV-2 for 2 h (MOI of 4), prior to medium removal. Cells were then incubated for 24h at 37 °C, prior to extraction of viral RNA and qRT PCR to determine the number of viral RNA genomes present in each sample as previously [17–19]. Data represent the mean + SEM (n ≥6). ** $p < 0.01$; **** $p < 0.0001$.

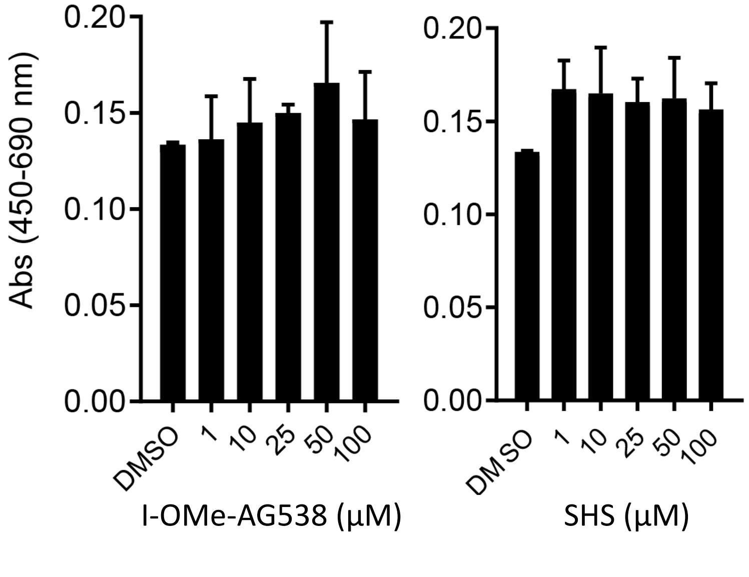

Figure S2. Lack of cytotoxicity of I-OMe-AG538 and SHS in Vero cells at concentrations effective in inhibiting flaviviruses. Vero cell viability was determined by addition of XTT reagent (Sigma-Aldrich) following compound treatments, as indicated. Cell survival is plotted relative to an untreated control. Data are the mean +/− SD of duplicate wells from a representative assay from a series of 2 experiments.

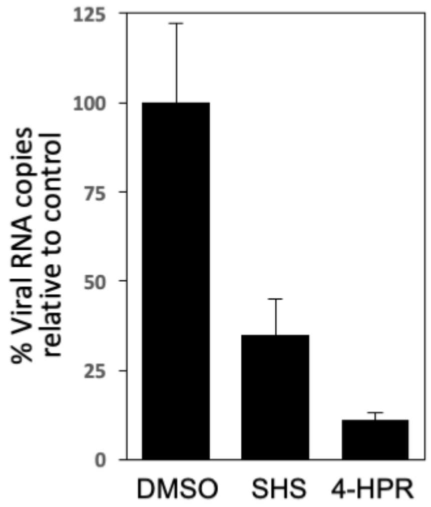

Figure S3. SHS has anti-DENV-2 activity even when added 6h post-infection. Vero cells were infected with DENV-2 for 2 h (MOI of 4), medium removed, and then treated 6h later without or with 10 μM SHS or 4-HPR (positive control) (in a final DMSO concentration of 0.2%) for 2 h, prior to removal of the medium and addition of fresh medium. Cells were incubated at 37 °C for 48 h post-infection, prior to extraction of viral RNA from the cells and qRT PCR to determine the number of intracellular viral RNA copies present in each sample [17–19]. Data represent the mean + SD of triplicate wells.
